# Mesenchymal Stem Cells cause Telomere Length Reduction of Molt-4 Cells via Caspase-3, BAD and P53 Apoptotic Pathway

**DOI:** 10.22088/IJMCM.BUMS.10.2.113

**Published:** 2021-09-01

**Authors:** Hamid Reza Heidari, Ezzatollah Fathi, Soheila Montazersaheb, Ayoub Mamandi, Raheleh Farahzadi, Soran Zalavi, Hojjatollah Nozad Charoudeh

**Affiliations:** 1 *Stem Cell Research Center, Tabriz University of Medical Sciences, Tabriz, Iran.*; 2 *Department of Pharmaceutical Biotechnology, Faculty of Pharmacy, Tabriz University of Medical Sciences, Tabriz, Iran.*; 3 *Department of Clinical Sciences, Faculty of Veterinary Medicine, University of Tabriz, Tabriz, Iran.*; 4 *Molecular Medicine Research Center, Tabriz University of Medical Sciences, Tabriz, Iran.*; 5 *Student Research Committee, Tabriz University of Medical Sciences, Tabriz, Iran.*; 6 *Hematology and Oncology Research Center, Tabriz University of Medical Sciences, Tabriz, Iran.*; 7 *Department of Anatomical Sciences, Faculty of Medicine, Tabriz University of Medical Sciences, Tabriz, Iran. *

**Keywords:** Mesenchymal stem cells, telomere length, hTERT, BAD, P53, caspase-3, apoptotic pathway

## Abstract

Mesenchymal stem cells (MSCs) as undifferentiated cells are specially considered in cell-based cancer therapy due to unique features such as multi-potency, pluripotency, and self-renewal. A multitude of cytokines secreted from MSCs are known to give such multifunctional attributes, but details of their role are yet to be unknown. In the present study, MSCs were cultured, characterized and co-cultured with Molt-4 cells as acute lymphoblastic leukemia cell line in a trans-well plate. Then, cultured Molt-4 alone and Molt-4 co-cultured with MSCs (10:1) were collected on day 7 and subjected to real time-PCR and Western blotting for gene and protein expression assessment, respectively. Ki-67/caspase-3 as well as telomere length were investigated by flow cytometry and real time-PCR, respectively. The results showed that MSCs caused significant decrease in telomere length as well as *hTERT* gene expression of Molt-4 cells. Also, gene and protein expression of BAD and P53 were significantly increased. Furthermore, the flow cytometry analysis indicated the decrease and increase of the Ki-67 and caspaspase-3 expression, respectively. It was concluded that MSCs co-cultured with Molt-4 cells could be involved in the promotion of Molt-4 cell apoptosis via caspase-3, BAD, and P53 expression. In addition, the decrease of telomere length is another effect of MSCs on Molt-4 leukemic cells.

Mesenchymal stem cells (MSCs) are multipotent undifferentiated cells which have a potential to differentiate into different cell types such as adipocytes, osteocytes, neuron like cells etc. (1). Other features of MSCs including self-renewal, plasticity, and non-immunogenic characteristics have led to consider them for cell-based therapy (2). The role of MSCs in some diseases such as cancer, blood disorders, heart failure, genetic and neurodegenerative diseases have been considered in more recent studies (3, 4). Hematologic malignancies have received more attention for cell transplantation with MSCs. The promoting and inhibiting effects of MSCs on cancer cells progression is one of the challenges associated with cell therapy (5, 6). Most studies confirmed the inhibitory effects of MSCs on tumor growth, however, only a few have shown stimulatory effects (7). The reason for this contradiction may be related to different types of tumor cells, the heterogeneity of MSCs population, and the number of injected cells (8). Because of these contradictions, further studies are suggested for investigating the precise bidirectional interaction between tumor cells and MSCs. In one study, Zhang and Zhang (2009) indicated that factors such as cytokines which are released from MSCs could inhibit the proliferation rate of chronic myeloid leukemia mononuclear cells (CML-MNCs) in patients (9). In contrast to the study mentioned above, Paino et al. (2018) demonstrated that SAOS2 osteosarcoma and MCF7 breast cancer cell lines are able to maintain adipose tissue-derived MSCs (ADSCs) in a stemnes state, and if these cells persist following surgery, they will most likely induce resident MSCs to boost tumor angiogenesis and proliferation (10). In more detail, the inhibitory/promoting effects of MSCs on tumor cells are driven by cytokines and other factors released from them. In this regard, Fonseka et al. (2012) pointed that interleukins (IL)-6 and IL-8 secreted from MSCs could inhibit the growth, and arrest the cell cycle of K562 cells as

CML cell line (11). 

Regarding the effect of MSCs on tumor cells, some signaling pathways such as mitogen-activated protein kinases (MAPK), AKT, glycogen synthase kinase (GSK)-3α/β, and extracellular signal-regulated kinase (ERK) 1/2 have been investigated. The aim of the present study was to investigate the effect of MSCs on telomere length (TL) and caspase-3 expression as an apoptotic marker of the Molt-4 cell line through BAD and P53 protein expression. For this purpose, Molt-4 cells cultured alone and co-cultured with MSCs (10:1) were collected on day 7, and subjected to TL assessment, human telomerase reverse transcriptase (*hTERT*) gene expression, and caspase-3 expression analyses. The protein expression of signaling pathways components involved in this process, including BCL2 associated agonist of cell death (BAD) and P53, were also evaluated.

## Materials and Methods

**Cell culture**

MSCs and Molt-4 cell line were purchased from Royan and Pasteur Institute (Tehran, Iran), respectively. Dulbecco’s Modified Eagle Medium (DMEM) with low glucose (Gibco Co. UK) and Roswell Park Memorial Institute (RPMI) (Gibco Co. UK) supplemented with 10% fetal bovine serum (FBS) (Gibco Co. UK) were used for MSCs and Molt-4 cell line culture, respectively (12). 

**Characterization of MSCs**

MSCs were characterized with cell surface markers investigation and multi-lineage differentiation potential to adipogenic and osteogenic cells as previously reported by Farahzadi *et al*. (2016) (13). For this purpose, MSCs from passage 4 were trypsinized, collected, and stained with specific antibodies against CD34, CD56, CD44, and CD90 (Santa Cruz Biotech- nology, CA, USA) for 30 min at 4 ℃. At the end of staining time, cells were washed using washing buffer (PBS supplemented with 3-5% FBS) and cell surface markers expression was quantified using FACS instrument. In the following, MSCs were seeded in the presence of adipogenic and osteogenic differentiation medium for 21 days. The components of differentiation medium were previously mentioned by Farahzadi et al. (2016) (13). Ethical consent was approved by Tabriz University of Medical Sciences, Tabriz, Iran (Ethic Code No: IR.TBZMED.VCR.REC. 1398. 215).

**Co-culture of MSCs and Molt-4 cell line**

MSCs of passage 4-6 were seeded at the density of 2×10^5 ^cells/well into trans-well insert with 0.4 µm microporous membrane (SPL Life Sciences Co, South Korea, Cat: 37306). After 12-16 h of culture, 1×10^6 ^Molt-4 cells/well was added to lower wells of trans-well plate. In this way two groups of cells were formed; control group (culture of Molt-4 alone) and experimental group (co-cultured Mol-4 and MSCs in a ratio of 10:1). At the end of co-culture at day 7, cultured Molt-4 cells alone and co-cultured Molt-4 cells with MSCs were subjected to genes, proteins, and caspase-3 expression assessment.

**Real-time PCR**

At the end of co-culture period, Molt-4 cells in both control and experimental groups were collected and mRNA expression of target genes *hTERT*, *BAD*, and *P53* were examined by real-time PCR. In more detail, total RNA was extracted using RNA extraction kit (Yekta Tajhiz Azma, Tehran, Iran). 2 μg RNA was used for the first strand cDNA synthesis according to manufacturer’s instructions. Corbett Rotor-Gene 6000 HRM was used for performing PCR reactions in a total volume of 20 μl containing forward and reverse primers (1 µM), SYBER Green master mix (Yekta Tajhiz Azma, Tehran, Iran) (2X), cDNA and H_2_O (14). The annealing temperature was 61 ˚C (*BAD*), 54 ˚C (*P53*), and 59 ˚C (*hTERT* and *β*-*actin*) for cDNA amplification. The final data was analyzed as Ct values in relation to β-actin Ct values by the 2^-ΔΔCT^ method (15, 16). Primers are presented in Table 1.

**Western blotting**

Protein expression of BAD and P53 was investigated by Western blotting. For this purpose, cells from control and experimental groups were collected and total cell proteins were extracted. Next, protein samples were electrophoresed on 12% polyacrylamide gels and transferred to a PVDF membrane. In the following, the membrane was incubated with primary antibodies against BAD and P53, and it was then incubated with corresponding secondary antibodies. The protein bands were detected with X-ray film and GAPDH was used as the internal control to normalize (17, 18).

**Absolute telomere length (aTL) measurement**

aTL was measured using real-time PCR as previously reported by Fathi et al. (2020) (19). Briefly, DNA was isolated from both control and experimental groups at the end of co-culture period and 20 ng/µl DNA was suitable for aTL measurement. Data obtained from real-time PCR for aTL was analyzed as kb/reaction and genome copies/reaction for telomere and single copy gene. The primers used for aTL measurment are listed in Table 2 (20).

**Table 1:** Primer sequences used for real time-PCR

**No.**	**Gene**	**Primer pair sequence (5'-3')**	**Product length (bp)**
NM_032989.3	*BAD*	ACTTCCTCGCCCGAAGAGC CTTCCCCTGCCCAAGTTCC	105
NM_001126118.1	*P53*	TCAGTCTACCTCCCGCCATAA AGTGGGGAACAAGAAGTGGAG	86
NM_001193376.3	*hTERT*	CAGCAAGTTTGGAAGAACCC GACATCCCTGCGTTCTTGG	98
NM_001101.4	*β-actin*	AAACTGGAACGGTGAAGGTG TATAGAGAAGTGGGGTGGCT	174

**Table 2:** Oligomers and characterizations used for absolute telomere length measurement

**Oligomer name**		**Oligomer sequence** **(5'-3')**	**Molecular** ** weight** **(MW)**	**Other calculations**		**Purification method**
To draw standard curve	Telomere standard	(TTAGGG)14	26667.2.	**Weight (g)**	2.6667×10^4^/6.02×10^23^= 0.44×10^-19^	HPLC
				**Number of molecules of oligomer in TEL standard A**	60×10^-12^/0.44× 10^-19 ^= 1.36×10^9^	
				**Amount of telomere sequence in TEL standard A (kbp)**	1.36 ×10^9^×84 = 1.18×10^8^	
	36B4 standard	5'CAGCAAGTGGGAAGGTGTAATCCGTCTCCACAGACAAGGCCAGGACTCGTTTGTACCCGTTGATGATAGAATGGG 3'	23268.1	**Weight (g)**	2.32681×10^4^/6.02×10^23^= 0.38×10^-19^	
				**Number of copies ** **of 36B4**	200×10^-12^/0.44× 10^-19^= 5.26×10^9^	
To calculate telomere length	Telo	Fwd: CGGTTTGTTTGGGTTTGGGTTTGGGTTTGGGTTTGGGTT Rev: GGCTTGCCTTACCCTTACCCTTACCCTTACCCTTACCCT				
	36B4	Fwd: CAGCAAGTGGGAAGGTGTAATCC Rev: CCCATTCTATCATCAACGGGTACAA				

**Apoptosis investigation by Ki-67/caspase-3 assay**

Ki-67/aaspase-3 assay was performed in co-cultured and non-co-cultured groups. In this regard, 20 ×10^4^ cells/well were washed with washing buffer and incubated with 0.2% Triton X-100 for 15 min. Next, the cells were stained with 5 μ Ki-67 antibody solution (BD Biosciences, USA, Cat: 556027) for 30 min and analyzed by flow cytometry. 

In addition, for the caspase-3 assay, cells from both groups were fixed and then permeabilized using fixation and permeabilization buffers (supplemented in kit), respectively. Washed cells were immediately stained using PE conjugated anti caspase (BD Biosciences, USA, Cat: 556027) and analyzed by flow cytometry.

**Statistical analysis**

The results were analyzed using t-test. The statistical significance was determined at P <0.05 by Graph Pad Prism version 6.01. 

## Results

**Characterization of MSCs with flow-cytometry and multi-lineage differentiation**

**Fig. 1:**
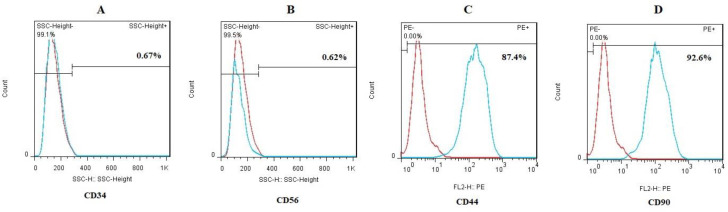
**Immunophenotypic characterization of MSCs; **The MSCs were negative for (A) CD34 (0.67%) and (B) CD56 (0.62%), and positive for (C) CD44 (87.4%) and (D) CD90 (92.6%). Also, isotype control is seen with red dots. Flow cytometry data was analyzed by FlowJo software (version 6.2)

**Fig. 2:**
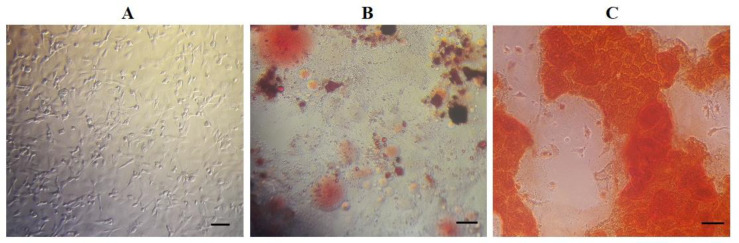
**Morphology of MSCs. **A) Fibroblast-like morphology of cells was seen (bar = 20 μm); B) Lipid vacuoles were stained by Oil-red O after abiogenesis (bar = 20 μm); C) Mineralized cell aggregates were stained by Alizarin red at the end of osteogenic differentiation (bar = 20 μm).

Flow cytometry analyzes (Figures 1A-D) show that MSCs expressed CD44 (87.4%) and CD90 (92.6%) as mesenchymal surface markers. However, hematopoietic surface markers CD34 (0.67%) and CD56 (0.62%) were not expressed in MSCs. In addition to flow-cytometry, adipogenic and osteogenic differentiation was investigated for multi-lineage differentiation potency of MSCs. As shown in Figure 2, lipid droplets as well as calcium deposits were stained with Oil-red O and Alizarin-red, respectively (13).

**Investigation of the gene and protein expression of BAD and P53 in Molt-4 cells following co-culture with MSCs**

For investigating the effect of cytokines secreted from MSCs on Molt-4 cells as acute lymphoblastic leukemia, mRNA and protein expression was examined by real time-PCR and Western blotting, respectively. 

**Fig. 3:**
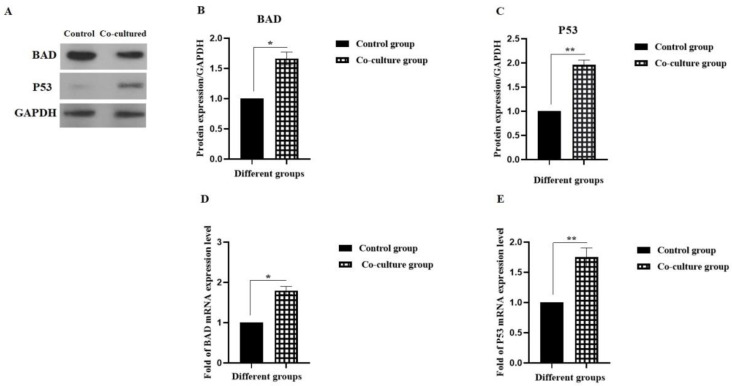
**Effect of MSCs on gene and protein expression of BAD and P53 in Molt-4 cell line. **A-C) The protein expression of BAD and P53 ; D and E) The mRNA expression levels of *BAD* and* P53*. *P < 0.05 and **P < 0.01

In this regard, BAD and P53 as apoptotic elements were investigated. As shown in Figures 3A-C, the protein expression levels of BAD and P53 was significantly increased by about 1.66 and 1.96 folds in the experimental group in comparison with the control group, respectively (*P <0.05 and **P < 0.01). In addition, the mRNA levels of *BAD* and *P53* were significantly increased by about 1.8 and 1.75 folds in the experimental group in comparison with the control group, respectively (Figs 3D and E) (*P <0.05 and **P < 0.01). 

**Investigation of aTL and** ***hTERT*** **gene expression following co-culture with MSCs**

**Fig. 4:**
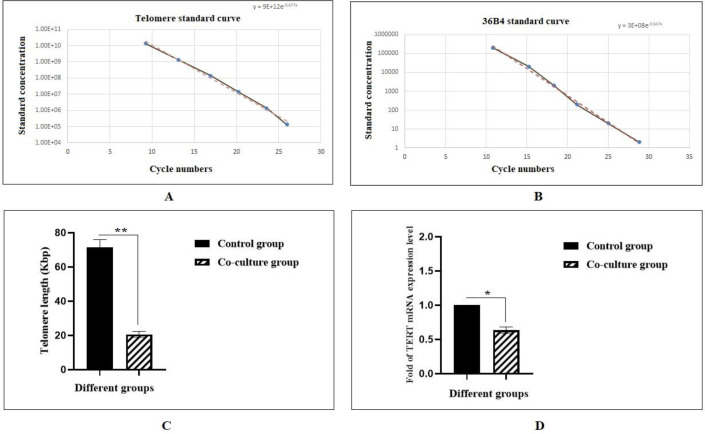
**Absolute telomere length measurement of Molt-4 cells following co-culture with MSCs.** A) The standard curve for calculating the length of telomere sequence per reaction tube; B) The standard curve for calculating genome copies using the 36B4 copy number; C) Telomere length (TL) of Molt-4 cells were compared to Molt-4 cells co cultured with MSCs. MSCs decreased significantly the TL of Molt-4 cells upon co-culture (**P < 0.01); D) Relative *hTERT* gene expression levels of Molt-4 cell line at the end of 7th day co-culture period with MSCs. MSCs decreased significantly the expression of *hTERT* gene in Molt-4 cell line (*P < 0.05).

Following the co-culture period, aTL was measured using real-time PCR. As shown in Figs 3 A-C, aTL was significantly decreased (20.63 Kbp) compared to the control group (71.61 Kbp) (**P<0.01). Also, the *hTERT* gene expression has decreased by about 0.68-fold in the experimental group in comparison with the control group (Figure 4D) (*P <0.05). 

**Investigation of cell apoptosis by Ki 67-casapase3 assessment following co-culture with MSCs**

The predominant effect of MSCs on inhibition of Molt-4 cell proliferation was detected by Ki-67 expression. 

**Fig. 5:**
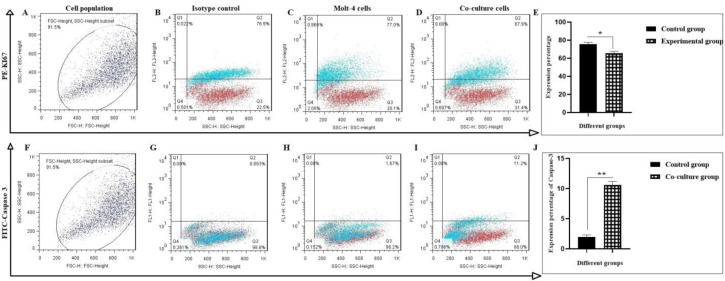
**Proliferation assay of Molt-4 cells following co-cultured with MSCs. **A-E) Harvested cells were evaluated with Ki-67 through flow cytometry; F-J) Flow cytometry analysis of caspase-3 in harvested cells. A and F are selected cell population; B and G are isotype control; C and H are control group; D and I are Molt-4 cells co-cultured with MSCs. Values are mean ± SD from independent experiments (*P < 0.05 and **P < 0.01, n = 3).

It was shown that co-culture condition attributed to the downregulation of Ki-67 in Molt-4 cells. At the end of co-culture period (7 days), the amount of Ki-67 in Molt-4 cells was 67.9% in comparison with control cells scored that contained 77.0% (Figures 5A-E) (*P < 0.05). Also, Molt-4 cells apoptosis was assessed following their co- culture with MSCs by caspase-3 investigation.

Data showed that caspase-3 level in the co-cultured group was increased 6.7-fold (Figurs 5F-J).

## Discussion

Hematologic malignancies are among the major causes of mortality throughout the world, and new therapeutic methods attract physicians and investigators. Stem cells present characteristics such as differentiation potential, self-renewal ability, etc. They are classified into two general groups of cells; somatic or adults and embryonic stem cells (21). Adult stem cells are multipotent undifferentiated cells with the multi-lineage differentiation potential. Among these, MSCs have received more attention in cell-based therapy (21). Currently, some diseases including heart failure, chronic wounds, liver disease, sepsis and respiratory diseases, and neurodegenerative diseases, are candidates for cell therapy (22). Recently, anticancer applications of MSCs have been widely considered. Stem cells can function as novel delivery aims by homing to and targeting both primary and metastatic tumor cells (23). There is abundant evidence that different types of MSCs could prevent tumor growth *in vitro*. Ganta *et al*., (2009) demonstrated that umbilical cord blood stem cells diminished mammary adenocarcinoma in a rat model (24). Furthermore, it was reported that intra-tumoral injection of ADSCs in a model of pancreatic adenocarcinoma inhibited tumor growth (25). Secchiero *et al*. (2010) showed that bone marrow (BM)-MSCs could abolish tumor growth in immune deficient mice bearing disseminated non-Hodgkin’s lymphoma xenografts (6). It has further been pointed that the ADSCs inhibited the *in vitro* proliferation rate of U251 glioma cells (26). Cetintas *et al*. (2014) indicated that BM-MSCs inhibited significantly the proliferation of ALL cell line CCRF-CEM via changes in the gene and protein expression levels of BAX, caspase 9, and P53 (27). The promoting effects of MSCs on cancerous cells were also been reported as contradictory results (28,29). These contradictory effects can be due to different cancer cells or different MSCs sources. Previous investigations have shown that MSCs affect cancer cells by releasing cytokines, chemokines, and growth factors (30). The results from the current study support the hypothesis that MSCs has inhibitory effects on ALL cell line Molt-4. The decreased and increased protein expression of Ki-67 and caspase3 might be the potential factor for this inhibition, respectively. The accurate role of different factors such as cytokines in leukemic cell survival is not yet well realized. Therefore, studying the cytokine and chemokine array is recommended in future investigations. On the other hand, telomerase expression followed by an increase in TL is critical for cell survival in cancer; thus, its important role as a target for cancer therapeutics is a topic that is widely considered. In most cancers, telomerase is activated to manipulate TL. Accordingly, reduced telomerase activity as well as TL, can be used as therapeutic targets to dominate cancers (31). Keller et al. (2009) reported that TL measured in peripheral blood of patients with CML correlates with disease stage, clinical prognostic scores, and response to treatment (32). In another study, Braig et al. (2014) demonstrated that telomerase-targeting strategy could alleviate the tumor promoting effect of BCR-ABL via inducing senescence in CML-like cells (33). The current results showed a significant relationship between *hTERT* gene expression and TL in Molt-4 cells co-cultured with MSCs. Since TL is regulated by the expression of the *hTERT* gene, there might be an association between the TL and *hTERT* expression. In addition to the mentioned items, many signaling pathways involved in cancer progression and apoptosis have been characterized; among these, the P53, BAD, and caspase3 pathways are important. 

Also, it was shown that upregulation of P53 and BAD in some cancer cells via downregulation of BCL-2 protein can lead to apoptosis (34, 35). In another study, Chen *et al.* (2017) pointed that the inhibition of telomerase activity can lead to cellular senescence via a P53-dependent mechanism (36). These results are in line with the results of the current study where overexpression of P53 was associated with decreasing TL and *hTERT* gene expression. These data suggest that the reduction in TL and *hTERT* gene expression was governed by the P53, BAD, and caspase3 signaling pathways. Without any ethical concerns, MSCs are easily obtained and cell therapeutic strategy using these cells seems to be a better choice for cancers. However, further researches are needed to use MSCs as clinical application.
